# Physiotherapeutic Approach Towards Sensory and Motor Recovery in a Patient With Lateral Mass Fixation: A Report of a Rare Case

**DOI:** 10.7759/cureus.60913

**Published:** 2024-05-23

**Authors:** Jaee P Kapre, Pallavi Harjpal, Komal S Mandhane, Ketki Kunjarkar

**Affiliations:** 1 Department of Neurophysiotherapy, Ravi Nair Physiotherapy College, Datta Meghe Institute of Higher Education and Research, Wardha, IND

**Keywords:** atlantoaxial joint, atlantoaxial subluxation, rehabilitation, physiotherapy, cervical myelopathy, c1-c2 lateral mass fixation

## Abstract

Atlantoaxial dislocations (AAD) are a diverse set of C1-C2 rotatory subluxations that include the inferior and superior axial facet articulations. C1-C2 segments are both covered by cranial-cervical ligaments, indicating that AAD would damage both joints. Whenever the posterior elements are missing or impaired, lateral mass screw fixation has replaced alternative posterior cervical fixation procedures as the preferred treatment for securing the sub-axial cervical spine. An increase in muscle tone, hyperreflexia, pathological reflexes, digit/hand clumsiness, and gait deviations caused by spinal cord compression at the cervical level are the most common clinical features. A 23-year-old female patient came with the chief complaint of weakness, tingling sensation, and numbness in both upper and lower limbs along with imbalance while walking. She had a history of falls which was managed conservatively. As the symptoms progressed, an MRI, a CT scan, and an X-ray of the neck were done to rule out the level of injury which revealed AAD, and the patient was operated on for C1-C2 lateral mass fixation. Post-operatively, the patient was referred to the physiotherapy department for further management. The patient's quality of life and daily functioning were positively affected after undergoing early intervention as measured by the Functional Independence Measure, Neck Disability Index, Berg Balance Scale, and Dynamic Gait Index.

## Introduction

Atlantoaxial subluxation is uncommon, and early detection and treatment are critical to achieving positive neurological results. Because of the specific biomechanics of these injuries, care for such traumatic unilateral atlantoaxial rotatory subluxation (AARS) differs; they frequently necessitate a personalised approach [1]. The currently accepted method for posterior cervical fusion uses lateral mass screw (LMS) fixation with plates or rods. Comparable post-operative segmental stability is provided by bilateral posterior cervical cages and lateral mass structures, both of which dramatically reduced the cervical range of motion [2]. A surgical procedure that is becoming more and more popular for treating atlantoaxial instability is the C1-C2 plate or rod and screw fixation. Excellent fusion rates are achieved with fixation using a plate or rod, a C1 or C2 screw, and structural bone grafting. The procedure is technically challenging and has a chance of vertebral artery, internal carotid artery, spinal cord, and hypoglossal nerve damage. A technique for C1-C2 fixation has been devised that involves bilaterally inserting C2 pedicle screws and rolling C1 titanium cable through the posterior arch of the atlas and the plate's cranial hole before fixing the C1-C2 plate [3]. Numerous aetiologies, such as trauma, infection, inflammation, or congenital defects, can cause atlantoaxial joint instability. A single piece of iliac crest bone graft was put between C1 and C2, and it was fastened with a steel wire in a sublaminar fashion according to Gallie's method of C1-C2 fusion. The medial facet line divides the lateral mass, which connects the superior and inferior articular processes, from the lamina. Injury to nearby nerve roots, vertebral arteries, or the spinal cord, facet joint violations, pseudoarthrosis, and screw fixation failure are the most common side effects of lateral mass fixation [4]. The posterior technique was first used to implant bilateral C1-C2 transarticular screws by Magerl and Seeman. The increased bony area for screw placement when compared to the Magerl method is one potential benefit of using the lateral bulk of the atlas for fixation [5].

A disorder known as cervical myelopathy is when the spinal cord is compressed at the cervical level of the spinal column, causing increase in muscle tone, exaggerated reflex, pathologic reflexes, clumsiness in the fingers and hands, and/or gait disruption [6]. Patients who are not treated may have significant paralysis and function loss. Upper extremity symptoms prevail in cervical myelopathy. These could include clumsy hands and a restricted capacity for fine motor activities including combing hair, holding small items, and buttoning shirts. Additionally, typical are radicular symptoms and neck aches. Surgery entails the decompression of the restricted region from the anterior or posterior and likely fusion [7]. And over 18 months of symptom duration, a reduction in cervical spine range of motion, and female gender are all indicators of a bad prognosis. Surgical decompression will help approximately 65% of individuals suffering from neck pain and radicular symptoms [8]. It has been demonstrated that cervical stenosis and myelopathy can be treated surgically, both anteriorly and posteriorly. It has been demonstrated that surgical procedures used to treat cervical stenosis and myelopathy, both anterior and posterior, improve patient outcomes [9].

Analgesic and anti-inflammatory medicines, muscle relaxants, physical therapy approaches, and orthoses are all examples of conservative treatment. Using some type of passive cervical manipulation, cervical collar or halter or skeletal traction, and halo immobiliser are all alternatives for realigning the neck in a decreased posture in physical therapy [10]. Cervical halter traction in the supine position for 24-48 hours, accompanied by mobility with orthotic immobilisation with active range of motion activities, is continued until free mobility is restored [11].

## Case presentation

Patient information

A 23-year-old female who was a student residing in Amravati came with chief complaints of weakness, tingling sensation, and numbness in both upper and lower limbs for four months, specifically more on the left side of the body. She also complained of imbalance while walking for three months. The patient was alright for four months, and she had a fall, which was managed conservatively. But later on, she slowly and gradually started realizing that there is a bilateral lower limb and upper limb weakness (the left side more than the right). She also has a previous operated history of hemorrhoidectomy, 15 days back. Then, she visited a private hospital where it was managed conservatively by medications. Then, the patient came to our neurosurgery outpatient department (OPD) with the complaints mentioned above, and she was suggested for a magnetic resonance imaging (MRI), computed tomography (CT) scan, and X-ray of the neck. The MRI revealed loss of cervical lordosis, and the CT scan revealed os odontoideum along with atlantoaxial dislocation (AAD). The neurophysician suggested a Philadelphia collar for neck stabilisation, and she was admitted to the neurosurgery ward on 09/09/22. Then, the patient was operated on 16/09/22 for C1-C2 lateral mass fixation. On 20/09/22, she was referred to physiotherapy for further management and rehabilitation, and after a few months, she was able to walk independently with good balance. The timeline of events is shown in Table 1.

**Table 1:** Timeline of events ICU: intensive care unit, L: left, R: right, OPD: outpatient department

Sr. no.	Date of events	Consultation	Findings
1	On admission	Neurosurgery OPD	Weakness, tingling sensation, and numbness in both upper and lower limbs (L>R), imbalance while walking
2	09/09/2022	Neurosurgery ward	Admitted for observation
3	10/09/2022	Neurosurgery ward	X-ray (anterior subluxation of the atlas over the axis vertebrae on the left side)
4	16/09/2022	Neurosurgery ICU	Post-surgery (for one day)
5	20/09/2022	Neurosurgery ward	Call for neurophysiotherapy

Clinical findings

The patient was evaluated while lying supine on a plinth. The vital indicators such as heart rate were increased to 78 beats/min; the respiratory rate was 17 breaths/min. On observation, the patient had an endomorphic build and was wearing a Philadelphia collar for neck stabilisation. The upper extremity was situated by the body's sides, with the forearm in pronation. In bilateral lower limbs, her hips were extended, abducted, and externally rotated with the knee in extension and the ankle in slight plantar flexion. There was the use of accessory muscles of respiration with the thoracoabdominal type of breathing due to a recent operation of the neck region. There was no evidence of oedema or pressure sores. On palpation, there were no noteworthy findings in the context of warmth, tenderness, and swelling. On examination, the higher mental functions were normal, i.e., the cognition of the patient was not impaired as the score was 29/30 on the Mini-Mental State Examination (MMSE). Her short- and long-term memory was intact. In special senses, only VIII (sensory) cranial was impaired. In the sensory examination, the superficial, deep, and cortical sensations were intact bilaterally over the upper and lower limbs along with the trunk. In the motor examination, there was weakness in both upper and lower limbs with truncal instability. Hoffman's sign and Romberg's sign were also positive. On manual muscle testing (MMT), her upper limbs had a grade of 3/5, and her lower limbs had a grade of 3+/5. Her deep tendon reflexes were normal. The neural tension test was positive for the median and radial nerve on the left side as shown in Table 2.

**Table 2:** Special test: ULTT ULTT: Upper Limb Tension Test

ULTT	Right	Left
ULTT 1	Negative	Positive
ULTT 2	Negative	Positive
ULTT 3	Negative	Positive
ULTT 4	Negative	Negative

Clinical diagnosis

**Figure 1:**
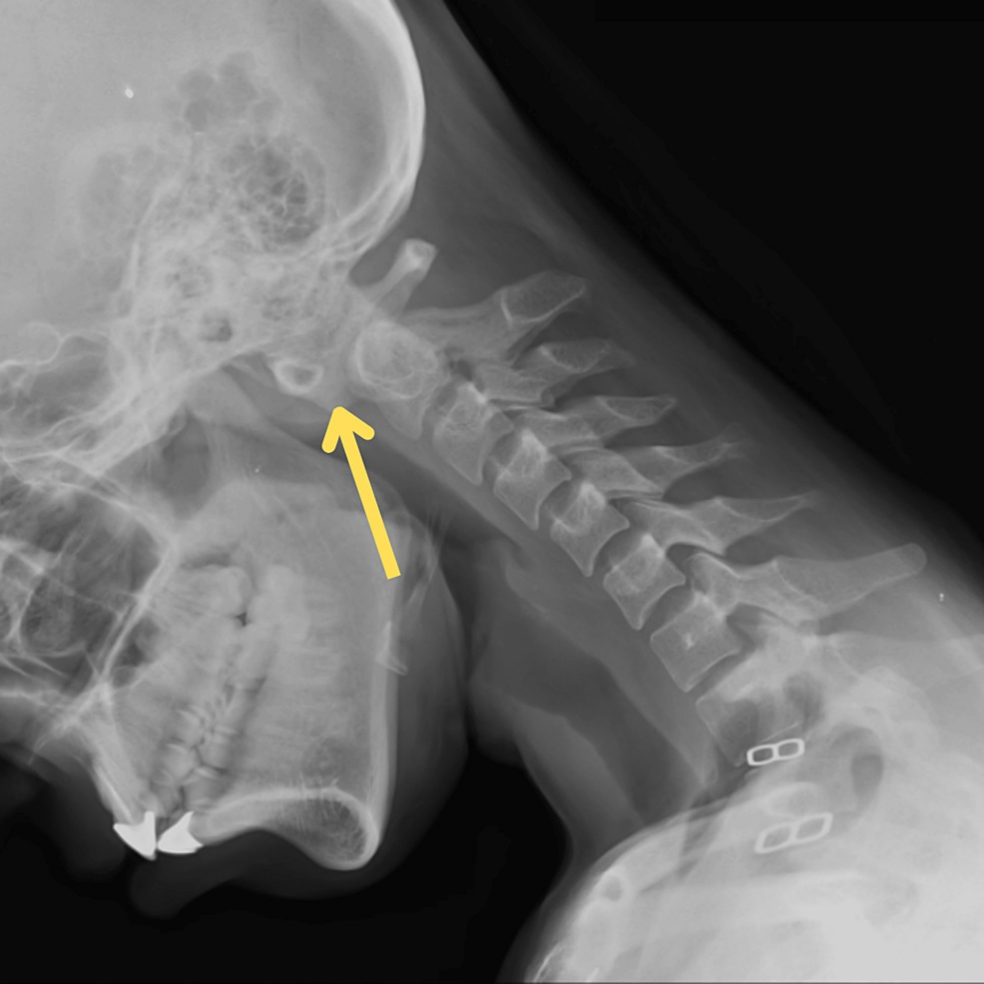
Anterior subluxation of the atlas over the axis vertebrae visible on X-ray of the cervical spine taken in flexion position

**Figure 2:**
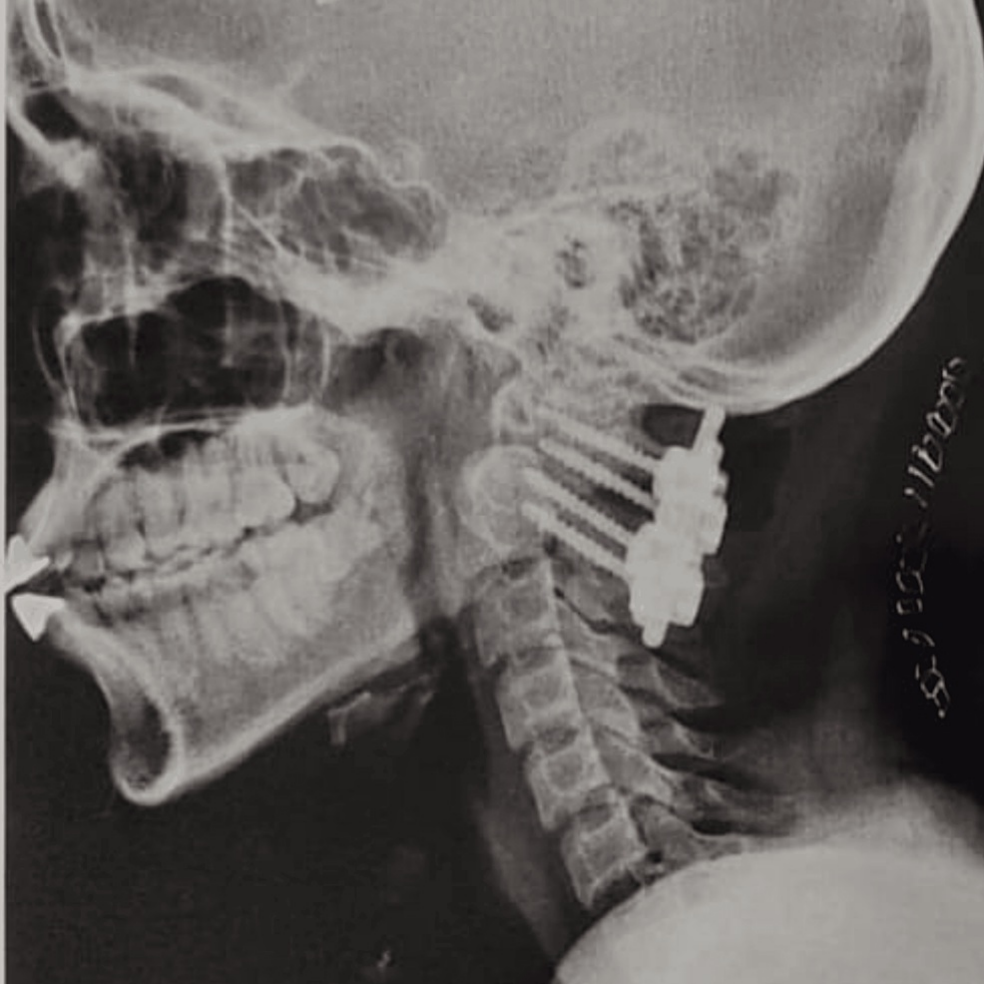
Post-operative C1-C2 lateral mass fixation

The patient received a cervical spine MRI examination, which demonstrated a decrease in cervical lordosis. CT scan showed os odontoideum and 6 mm anterior subluxation of the atlas over the axis vertebrae on the left side. X-ray showed anterior subluxation of the atlas over the axis vertebrae (with red arrow) on the left side in Figure 1. Figure 2 shows the post-operative X-ray of the patient's cervical region.

Physiotherapy intervention

**Figure 3:**
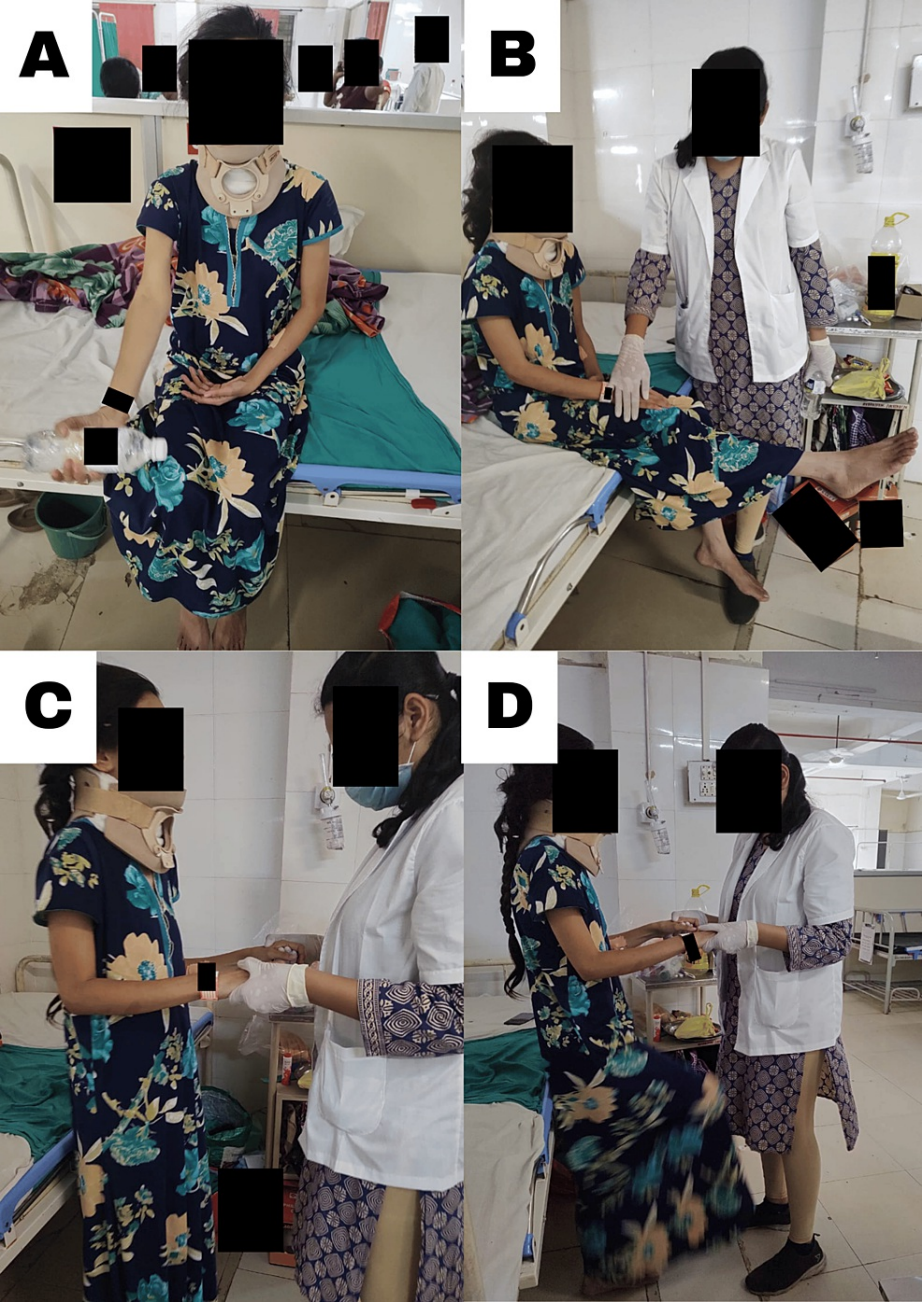
Various exercises performed by the patient (A) Upper limb strengthening exercises. (B) Dynamic quadriceps strengthening. (C) Balancing training. (D) Alternative leg raising

The patient was properly informed of the benefits of physiotherapy, and the procedure was quickly presented to her. After four days of operation, physiotherapy was started, and the patient was shown visually and orally how to do the following activities which are shown in Table 3 and Figure 3. The following treatment procedure was administered once a day for 10 repetitions of one set with enough rest. An appropriate amount of intervals was also offered to the patient to avoid weariness.

**Table 3:** Physiotherapy intervention L: litre, kg: kilogram, PNF: Proprioceptive Neuromuscular Facilitation

Problem list	Goals	Interventions
Both upper and lower limb weakening	To help boost upper and lower limb strength	Bilateral upper limb strengthening training (1/2 L water bottle and progress to 1 L). Bilateral lower limb strengthening training (1/2 kg weight cuff and progress to 1 kg weight cuff)
Tingling and numbness in both upper and lower limbs	To reduce tingling	Neural mobilization for the upper limb and lower limb (until the symptoms subsided)
Restricted cervical range of motion	To improve the cervical ranges	Active cervical range of motion exercises, neck isometric exercises, and chin tucks (muscle energy technique was used to train the neck muscles)
Reduced bed transfers	To facilitate transfers	Supine-to-side lying training, side lying-to-sitting training, and sitting-to-standing training
Decreased mobility	To achieve mobility	Swiss ball, trunk PNF with upper limb movements, transition training, and rolling facilitation
Impaired balance	To regain static and dynamic balance	Perturbation and balance training, weight shifting, dynamic standing balance with perturbations, dynamic standing balance with reach-outs, tandem walking, asking the patient to pick up the objects from the floor, and trunk rotation exercises
Impaired gait	For gait training	Parallel bar training exercises, spot marching, and obstacle walking
Tendency to attain neck flexion posture	For ergonomic modification	Ergonomic advice, cervical range of motion exercises, chin tucks, and stretches

Follow-up and outcome measures

**Figure 4:**
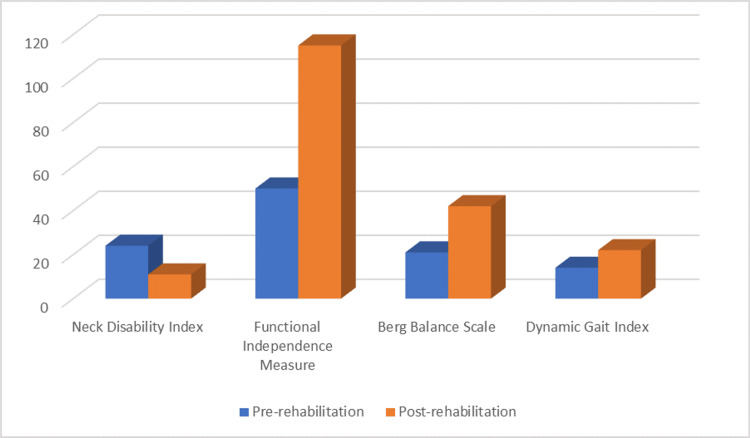
Pre-rehabilitation and post-rehabilitation outcome measures

There was an improvement in the MMT score, and the outcome measures, i.e., Functional Independence Measure, Neck Disability Index, Berg Balance Scale, and Dynamic Gait Index, are shown in Table 4, Table 5, and Figure 4.

**Table 4:** MMT score pre- and post-rehabilitation MMT: manual muscle testing

MMT	Pre-rehabilitation		Post-rehabilitation	
	Right	Left	Right	Left
Shoulder	3+/5	2/5	5/5	4/5
Elbow	3/5	3/5	4/5	4/5
Wrist	3+/5	3/5	5/5	4/5
Hip	3/5	2/5	4/5	4/5
Knee	3/5	2/5	4/5	4/5
Ankle	3/5	3/5	4/5	4/5

**Table 5:** Outcome measures

Outcome measures	Pre-rehabilitation	Post-rehabilitation
Neck Disability Index	24/50 (moderate disability)	11/50 (mild disability)
Functional Independence Measure	50	115
Berg Balance Scale	21/56	42/56
Dynamic Gait Index	14/24	22/24

## Discussion

In the majority of patients, rigid fixation with transarticular screws has been shown to be a secure and reliable technique for C1-C2 stabilisation. In comparison to wire/cable fixation, rigid fixation of C1-C2 through transarticular screw implantation provides rapid stability and has superior biomechanical qualities [12]. Adults occasionally experience the trauma of subluxation of the atlantoaxial joint, but there is currently no accepted standard of care. Important static stabilisers, the transverse and alar ligaments, stop the anterior translation of the C1 on the C2 vertebrae [13]. The cervical collar must be worn for at least eight hours continuously per day for three months, with usage time decreasing throughout the course of treatment, along with general rest when doing tasks [14]. When there is no neurological damage following a cervical injury, the patient's prognosis is worse in the short to medium term due to the delayed diagnosis and initial stabilisation. Reduction and early fusion are definite treatments to stop neurological problems from developing. The primary objectives of the physiotherapy programme are to increase ranges of motion at the cervical spine and shoulder, increase strength, enhance hand functions, improve balance and coordination, and avoid any additional deformity [15].

The congenital fusing of the atlas and the occipital bone is termed atlantooccipital fusion. It is one of the uncommon skeletal variants of the craniocervical area, occurring in 0.12-0.72% of the population [16]. Atlantoaxial Rotatory Dislocations (AARDs) are a diverse set of post-traumatic disorders that are common in children but uncommon in adults [17]. One of the physiotherapeutic rehabilitative uses of task-specific balance rehabilitation is perturbation-based balance training (PBT). Neural mobilization is proven to be effective to reduce tingling in such cases. The muscle energy technique prevents muscle fatigue and enables the strengthening of the muscle to produce better outcomes. When exposed to repeated perturbations, the neuromotor system may be encouraged to acquire the necessary neurophysiological modifications and sensorimotor skills to prevent collapsing [18].

## Conclusions

C1 lateral mass fixation is a safe alternative for upper cervical fixation with several potential advantages versus other techniques. Our study emphasises the improvement of patients based on physiotherapeutic interventions that are planned to improve the functional independence and quality of life of the patient. Rehabilitation of such patients requires time to recover, but with proper week-wise intervention, the patient can return to her normal daily life. Neurorehabilitation has different approaches, due to which the recovery of the patient can be fastened. In this case, after the surgery, physiotherapy plays a vital role in the patient's recovery.
